# Taxonomy of the *Colocasiomyia gigantea* species group (Diptera, Drosophilidae), with descriptions of four new species from Yunnan, China

**DOI:** 10.3897/zookeys.406.7176

**Published:** 2014-04-30

**Authors:** Nan-Nan Li, Masanori J. Toda, Zhao Fu, Ji-Min Chen, Su-Hua Li, Jian-Jun Gao

**Affiliations:** 1 Laboratory for Conservation and Utilization of Bioresources, Yunnan University, Kunming, Yunnan 650091, China; 2 School of Forestry, Southwest Forestry University, Kunming, Yunnan 650224, China; 3 Hokkaido University Museum, Hokkaido University, Sapporo, Japan

**Keywords:** Adaptation, aroid, character-based barcoding, cohabitation, genetic distance, integrated taxonomy, *Rhaphidophora* clade

## Abstract

Species of the genus *Colocasiomyia* de Meijere feed/breed on inflorescences/infructescences of the plants from the families Araceae, Arecaceae and Magnoliaceae. Although most of them utilize plants from the subfamily Aroideae of Araceae, three species of the recently established *C. gigantea* species group make use of plants of the subfamily Monsteroideae. We describe four new species of the *gigantea* group found from Yunnan, China: *Colocasiomyia longifilamentata* Li & Gao, **sp. n.**, *C. longivalva* Li & Gao, **sp. n.**, *C. hailini* Li & Gao, **sp. n.**, and *C. yini* Li & Gao, **sp. n.** The species delimitation is proved in virtue of not only morphology but also DNA barcodes, i.e., sequences of the partial mitochondrial *COI* (cytochrome *c* oxidase subunit I) gene. Some nucleotide sites with fixed status in the alignment of the *COI* sequences (658 sites in length) are used as “pure” molecular diagnostic characters to delineate species in the *gigantea* group.

## Introduction

To date, as many as 90 species (of them only 25 species described) have been found in the genus *Colocasiomyia* de Meijere, 1914. All these species visit and breed on flowers of the families Araceae, Arecaceae or Magnoliaceae (Sultana et al. 2006, Fartyal et al. 2013, Toda unpublished data). So far, six species groups, i.e., *baechlii*, *cristata*, *toshiokai*, *crassipes*, *zeylanica* and *gigantea* (Okada 1990, Sultana et al. 2002, 2006, Fartyal et al. 2013), have been established in this genus.

The *gigantea* group was recently erected by Fartyal et al. (2013) for two species [*Colocasiomyia gigantea* (Okada, 1987) and *Colocasiomyia scindapsae* Fartyal & Toda, 2013] from Southeast Asia and one species (*Colocasiomyia rhaphidophorae* Gao & Toda, 2013) from China (Table 1). Host plants of these species belong to the *Rhaphidophora* clade of the subfamily Monsteroideae of True Araceae, according to the most recent phylogenetic studies of the Araceae (Cusimano et al. 2011, Nauheimer et al. 2012). On the other hand, the other species groups utilize either the subfamily Aroideae or the other families. Fartyal et al. (2013) investigated the reproductive ecology of the three species, and revealed interesting ecological and morphological adaptations of the flies to the host plants. In addition, Fartyal et al. (2013) conducted a cladistic analysis of 70 morphological characters of 34 *Colocasiomyia* species, covering all the six species groups of the genus. Their results lent essential support to the monophyly of the *gigantea* group, placing it as the sister clade to the *cristata* species group.

**Table 1.** Summary of the species of the *Colocasiomyia gigantea* species group.

Species name	Distribution	Host plant	Reference
*Colocasiomyia gigantea* (Okada, 1987)	Java, Indonesia; Solomon Is.	*Epipremnum pinnatum* (L.) Engle	Fartyal et al. (2013)
*Colocasiomyia scindapsae* Fartyal & Toda, 2013	Sabah, Malaysia	*Scindapsus coriaceus* Engler	Ditto
*Colocasiomyia rhaphidophorae* Gao & Toda, 2013	Xishuangbanna, Yunnan, China	*Rhaphidophora hookeri* Schott	Ditto
	Pu’er, Yunnan, China	*Rhaphidophora decursiva* (Roxb.) Schott	Present study
*Colocasiomyia longifilamentata* sp. n.	Baoshan and Pu’er, Yunnan, China	Ditto	Ditto
*Colocasiomyia longivalva* sp. n.	Ditto	Ditto^a^	Ditto
*Colocasiomyia hailini* sp. n.	Ditto	Ditto	Ditto
*Colocasiomyia yini* sp. n.	Ditto	Ditto	Ditto

^a^ To be confirmed by further investigation (for details, see the “Remarks” section in the description of *Colocasiomyia longivalva* sp. n.)

Our recent field surveys in Yunnan Province, China brought new, insightful information on the evolution of flower-breeding habits in the *gigantea* group. We found four new species of this group visiting inflorescences of *Rhaphidophora decursiva* (Roxb.) Schott (Table 1); at least three of them were found breeding on inflorescences/infructescences of this plant. In Pu’er (central-southern part of Yunnan), *Colocasiomyia rhaphidophorae* cohabited with the above-mentioned three new species on inflorescences/infructescences of *Rhaphidophora decursiva*. Thus, the Chinese members of the *gigantea* group are mostly sympatric and overlapping in host plant selection with each other (cohabitation), in contrast to the allopatry and monopolization of host plant in the other members, *Colocasiomyia gigantea* and *Colocasiomyia scindapsae*.

The four new species of the *gigantea* species group are described here, based on species delimitation in virtue of morphological and molecular (DNA sequences of the mitochondrial cytochrome *c* oxidase subunit I gene, *COI*, as the DNA barcoding marker) characters.

## Materials and methods

### Materials

Table 2 shows the fly samples/specimens involved in the present study. Most of them were collected from the field in southwestern China, Malaysia and Indonesia. Some were reared from inflorescences/infructescences of host plants; after dissection of inflorescences/infructescences under a stereoscopic microscope in laboratory, the fly eggs were isolated and transferred into Petri dishes with decayed pistils as food and then reared at 25 °C in an incubator until adults emerged.

**Table 2.** Specimens of *Colocasiomyia* species used for DNA barcoding analysis.

Species	Voucher #/GenBank accession number	Collection site^d^
*Colocasiomyia sulawesiana*	Lot055 (DNA)/KJ700880^a^	A
*Colocasiomyia colocasiae*	Lot072 (DNA)/KJ700879^a^	B
*Colocasiomyia xenalocasiae*	001627/KJ700881	C
*Colocasiomyia gigantea*	Lot150 (DNA)/KJ700882^a^; 001444/KJ700883^a^; 001445/KJ700884^a^; 001449/KJ700885^a^	D
*Colocasiomyia scindapsae*	Lot036 (DNA)/KJ700886^a^; Lot037 (DNA)/KJ700887^a^; 001186/KJ700888^a^; 001508/KJ700889^a^; 001509/KJ700890^a^; 001510/KJ700891^a^	B
*Colocasiomyia rhaphidophorae*	000323/KJ700892^b^; 001137/KJ700893^a^; 001138/KJ700894^a^; 001512^c^/KJ700895^a^; 001513^c^/KJ700896^a^; 001447^c^/KJ700897^a^	E
	001514^c^/KJ700898^a^; 001516^c^/KJ700899^a^; 001525^c^/KJ700900^a^; 001530^c^/KJ700901^a^	C
*Colocasiomyia longifilamentata* sp. n.	000349/KJ700902^b^; 000350/KJ700903^b^; 000353/KJ700904^b^	F
	001515^c^/KJ700905^a^; 001523^c^/KJ700906^a^; 001526–1529^c^/KJ700907–0910^a^; 001531–1533^c^/KJ700911–0913^a^; 001576^c^/KJ700914^a^; 001577^c^/KJ700915^a^	C
*Colocasiomyia longivalva* sp. n.	000082/KJ700916^b^; 000086/KJ700917^b^	F
*Colocasiomyia hailini* sp. n.	000355–0358/KJ700918–0921^b^; 001298–1301^c^/KJ700922−0925^a^	F
	001448^c^/KJ700926^a^; 001450^c^/KJ700927^a^; 001517/KJ700928^a^	C
*Colocasiomyia yini* sp. n.	000160/KJ700929^b^; 000364/KJ700930^a^; 001185^c^/KJ700931^a^; 001194/KJ700932^a^; 001302^c^/KJ700933^a^; 001312^c^/KJ700934^a^	F
	001586^c^/KJ700935^a^	C

^a^ PCR/sequencing using the primers of Folmer et al. (1994);

^b^ PCR/sequencing using the primers of Hebert et al. (2004);

^c^ Adults obtained by laboratory rearing;

^d^ Collection sites: A, Enrekang, South Sulawesi, Sulawesi, Indonesia; B, Park Headquarters, Mt. Kinabalu, Sabah, Malaysia; C, Yixiang, Pu’er, Yunnan, China; D, Bogor Botanical Garden, West Java, Indonesia; E, Menglun, Mengla, Xishuangbanna, Yunnan, China; F, Baihualing, Longyang, Baoshan, Yunnan, China

### Morphological observation

We followed the same method as in Fartyal et al. (2013) for the observation of external morphology, measurement of morphometric characters and preparation of dissected organs. The male and female terminalia and cibarium of the new species were microphotographed using a DinoLite Digital Eyepiece Camera and drawn according to these digital pictures using CORELDRAW® X4 (Corel Corporation). Fine structures of foreleg (tibia, 1st and 2nd tarsomeres) and oviscapt were microphotographed for all the seven species of the *gigantea* group using a HITACHI TM3000 tabletop scanning electron microscope (SEM). We followed McAlpine (1981) for the morphological terminology, and Zhang and Toda (1992) for the definitions of measurements and indices. New type specimens were deposited in Kunming Natural History Museum of Zoology, Kunming Institute of Zoology, Chinese Academy of Sciences (KIZ) and Hokkaido University Museum, Hokkaido University, Sapporo, Japan (SEHU).

### DNA barcoding

A total of 54 individuals representing all the three known and four morphologically identified, putatively new species (Table 2) were subjected to DNA sequencing of the *COI* barcode fragments (Hebert et al. 2003). In addition, three more species, *Colocasiomyia colocasiae* (Duda, 1924), *Colocasiomyia sulawesiana* Okada & Yafuso, 1989 and *Colocasiomyia xenalocasiae* (Okada, 1980) from the sister *cristata* group were included, for comparison of sequences. DNA was extracted using small piece(s) of abdominal tissue or the right hindleg of a single adult using the TIANamp® Genomic DNA Kit. The primer pair used for the PCR and sequencing of the*COI* fragment was either that designed by Folmer et al. (1994, LCO1490: 5’- GGTCAA CAAAT CATAA AGATA TTGG -3’, HCO2198: 5’- TAAAC TTCAG GGTGA CCAAA AAATC A -3’) or that by Hebert et al. (2004, LepF1: 5’- ATTCA ACCAA TCATA AAGAT ATTGG -3’, LepR1: 5’- TAAAC TTCTG GATGT CCAAA AAATC A -3’). The 20 µl PCR reaction volume contains 0.1 µl *TaKaRa Ex Taq*® (5 U/µl), 0.4 µl of each primer (10 µM), 2 µl 10× *Ex Taq* Buffer (Mg^+^ Plus), 1.6 µl dNTP mixture (2.5 mM for each), 15 µl ddH_2_O and 0.5 µl of template DNA. The amplification of the *COI* fragments was initiated by 3-min pre-denaturation at 95 °C, followed by 35 thermocycles of 40 s of denaturation at 94 °C, 50 s of annealing at 46 °C, and 1 min of extension at 72 °C, ended by a 5 min of post-extension at 72 °C. The PCR products were purified and then subjected to DNA sequencing on an ABI® 3730 DNA Analyser.

A total of 57 *COI* sequences (54 of the *gigantea* group and three of the *cristata* group) were determined. The sequences were edited in the SEQMAN module of the DNASTAR package (DNASTAR Inc. 1996), and aligned in MEGA5 (Tamura et al. 2011). Then the inter- and intraspecific genetic distances were calculated for the species of the *gigantea* group using the Kimura 2-parameter (K2P) model in MEGA5. In addition, we also conducted a character-based species barcoding. Since there were end gaps (missing data) in some of the sequences of each species, the end sites with an overlapping of less than three (two for *Colocasiomyia longivalva*) sequences were excluded. The sites being fixed within a species but differing from the other species (including the three species of *cristata* group) were manually selected as diagnostic sites (i.e., “pure” diagnostics, Sarkar et al. 2002, DeSalle et al. 2005), for each species.

## Results

### DNA barcoding

The alignment of the 57 *COI* sequences spanned 658 nucleotide sites in length, with 184 variable sites, among which 160 were parsimony informative. For the inter- and intraspecific K2P distances see Table 3. The largest intraspecific K2P distance in the *gigantea* group was found in *Colocasiomyia scindapsae* (= 0.0102), while the smallest interspecific one was found between *Colocasiomyia rhaphidophorae* and *Colocasiomyia longifilamentata* (= 0.0135). This implies that the “barcoding gap” (Meyer and Paulay 2005) is too narrow, only 0.0033, to validate the distance-based species delimitation in the *gigantea* group.

**Table 3.** Intra- and interspecific K2P distances (minimum–maximum) in the *Colocasiomyia gigantea* species group.

Species	*N*^a^	Intraspecific distance	Interspecific distance					
			*Colocasiomyia gigantea*	*Colocasiomyia scindapsae*	*Colocasiomyia rhaphidophorae*	*Colocasiomyia longifilamentata* sp. n.	*Colocasiomyia longivalva* sp. n.	*Colocasiomyia hailini* sp. n.
*Colocasiomyia gigantea*	4	0–0.0058						
*Colocasiomyia scindapsae*	6	0–0.0102	0.1027–0.1236					
*Colocasiomyia rhaphidophorae*	10	0–0.0054	0.1056–0.1148	0.0978–0.1156				
*Colocasiomyia longifilamentata* sp. n.	14	0–0.0099	0.1134–0.1259	0.0972–0.1088	0.0135–0.0282			
*Colocasiomyia longivalva* sp. n.	2	0–0	0.1345–0.1370	0.0930–0.1046	0.0793–0.0860	0.0815–0.0834		
*Colocasiomyia hailini* sp. n.	11	0–0.0069	0.1099–0.1179	0.1017–0.1230	0.0939–0.1038	0.0978–0.1086	0.1079–0.1125	
*Colocasiomyia yini* sp. n.	7	0–0.0034	0.1440–0.1483	0.1425–0.1664	0.1211–0.1359	0.1367–0.1484	0.1431–0.1468	0.0674–0.0758

^a^ Number of sequences

**Figure 1.**
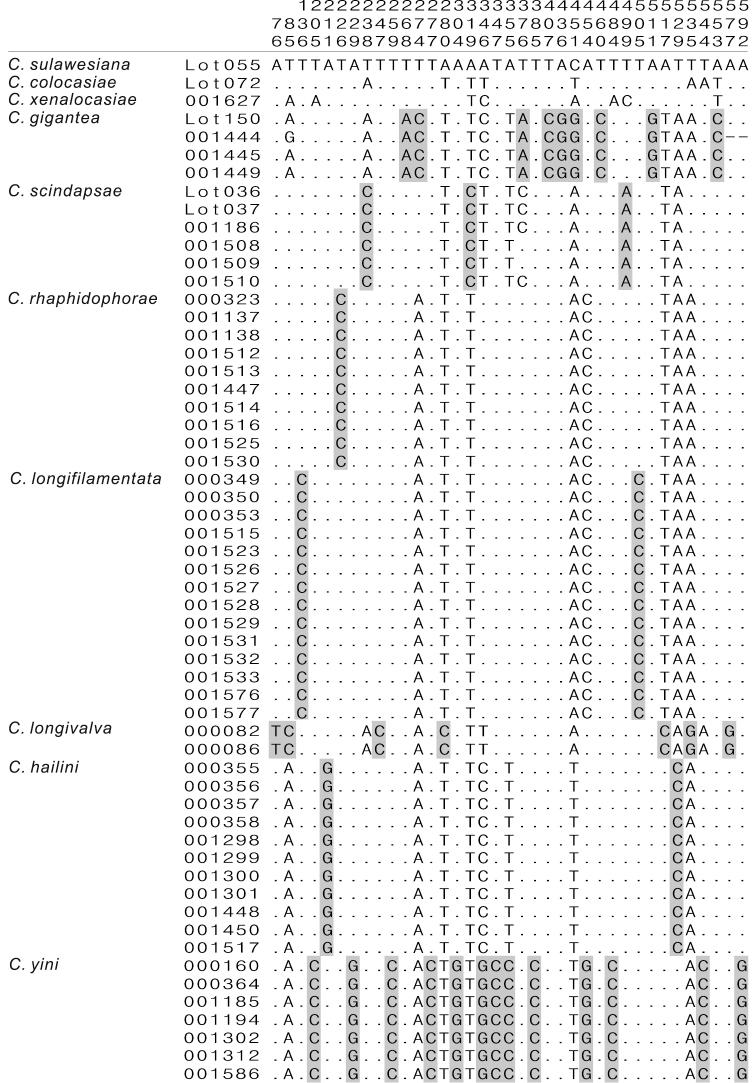
Diagnostic nucleotide sites in the alignment of *COI* sequences of the *Colocasiomyia gigantea* group. Numbers at the top show the positions of the sites in the *COI* alignment (658-bp in length). Shaded sites are diagnostic for each species. Hyphens (-) indicate end missing data.

Fig. 1 shows nucleotides at the sites where “pure” diagnostics for any species of the *gigantea* group are included. At least one diagnostic site was recognized for each species. For example, the site 226 is diagnostic for *Colocasiomyia rhaphidophorae*: this site has a fixed status of C (Cytosine) in this species, but T (Thymidine) in the other species. The sites 136 and 505 (both with fixed status of C) are diagnostic for *Colocasiomyia longifilamentata*.

## Taxonomy

### *Colocasiomyia gigantea* species group Fartyal et al. (2013).

**Included species.** *Colocasiomyia gigantea* (Okada, 1987); *Colocasiomyia rhaphidophorae* Gao & Toda in Fartyal et al. 2013; *Colocasiomyia scindapsae* Fartyal & Toda in Fartyal et al. 2013; *Colocasiomyia longifilamentata* Li & Gao, sp. n.; *Colocasiomyia longivalva* Li & Gao, sp. n.; *Colocasiomyia hailini* Li & Gao, sp. n.; *Colocasiomyia yini* Li & Gao, sp. n.

**Diagnosis** (Fartyal et al. 2013). Foreleg second tarsomere with 6–11 heavily sclerotized, nearly equally-sized pegs in 2 rows on ventro-apical elongation (Figs 2–15). Epandrium with very large, lobe-like apodeme on antero-subdorsal to -ventral margin (Figs 24, 30, 38, 45). Lateral lobes of oviscapt fused to each other only apically, with large patch of dense, distinct warts on basal half (Figs 16–22, 28, 36, 43, 50).

**Figures 2–22.**
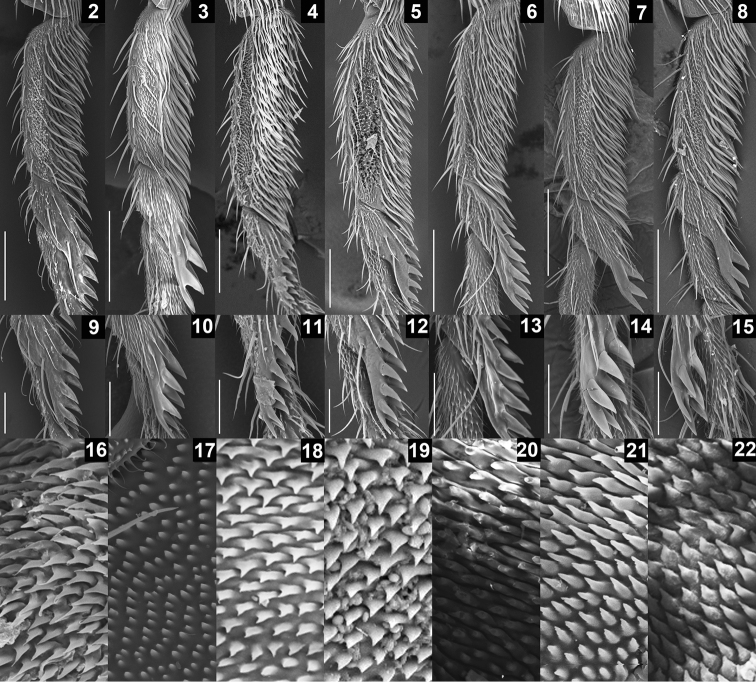
SEM photographs showing leg and oviscapt fine structures in the *Colocasiomyia gigantea* species group. Foreleg tarsomeres I and II (**2–8**), pegs on foreleg tarsomere II (**9–15**) and warts on basal part of lateral lobe or basal membrane of oviscapt (**16–22**) of *Colocasiomyia gigantea* (**2, 9, 16**), *Colocasiomyia scindapsae* (**3, 10, 17**), *Colocasiomyia rhaphidophorae* (**4, 11, 18**), *Colocasiomyia longifilamentata* sp. n. (**5, 12, 19**), *Colocasiomyia longivalva* sp. n. (**6, 13, 20**), *Colocasiomyia hailini* sp. n. (**7, 14, 21**) and *Colocasiomyia yini* sp. n. (**8, 15, 22**). Scale line = 0.1 mm in 2–8, 0.05 mm in **9–15.** Figures **16–22** are in the same magnification, with the width corresponding to 30 µm.

**Figures 23–28.**
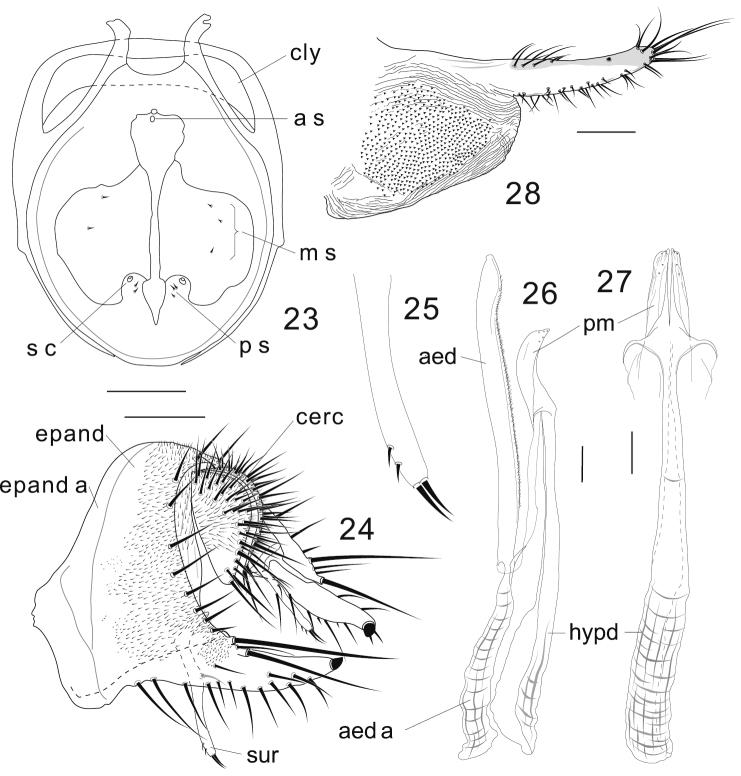
*Colocasiomyia longifilamentata* Li & Gao, sp. n. Adult male and female (paratypes) from Baihualing, Yunnan, China: **23** Cibarium and clypeus (dorsal view) **24** periphallic organs (posterolateral view) **25** apical part of surstylus **26** phallic organs (lateral view) **27** hypandrium and parameres (ventral view) **28** oviscapt (lateral view). Abbreviations: aed = aedeagus, aed a = aedeagal apodeme, a s = anterior sensilla, cerc = cercus, cly = clypeus, epand = epandrium, epand a = epandrial apodeme, hypd = hypandrium, m s = medial sensilla, ps = posterior sensilla, pm = paramere, s c = sensilla campaniformia, sur = surstylus. Scale lines = 0.1 mm.

**Figures 29–36.**
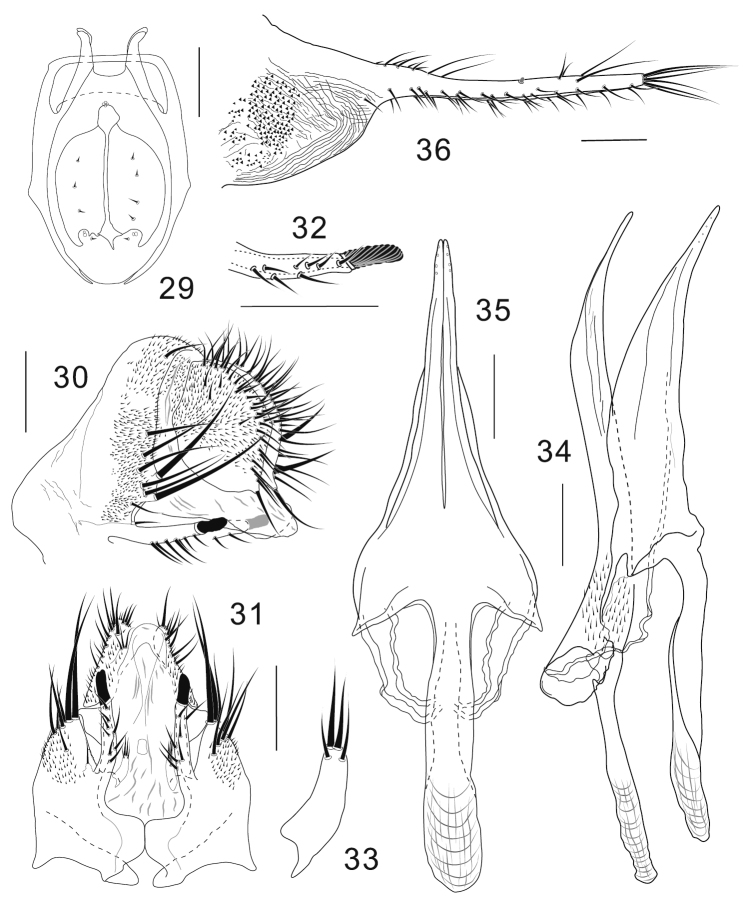
*Colocasiomyia longivalva* Li & Gao, sp. n. Adult male and female (paratypes) from Baihualing, Yunnan, China: **29** Cibarium and clypeus (dorsal view) **30** periphallic organs (posterolateral view) **31** periphallic organs (ventral view) **32** apical part of epandrial ventral lobe (ventral view) **33** surstylus (ventral view) **34** phallic organs (lateral view) **35** hypandrium and parameres (ventral view) **36** oviscapt (lateral view). Scale lines = 0.1 mm.

**Figures 37–50.**
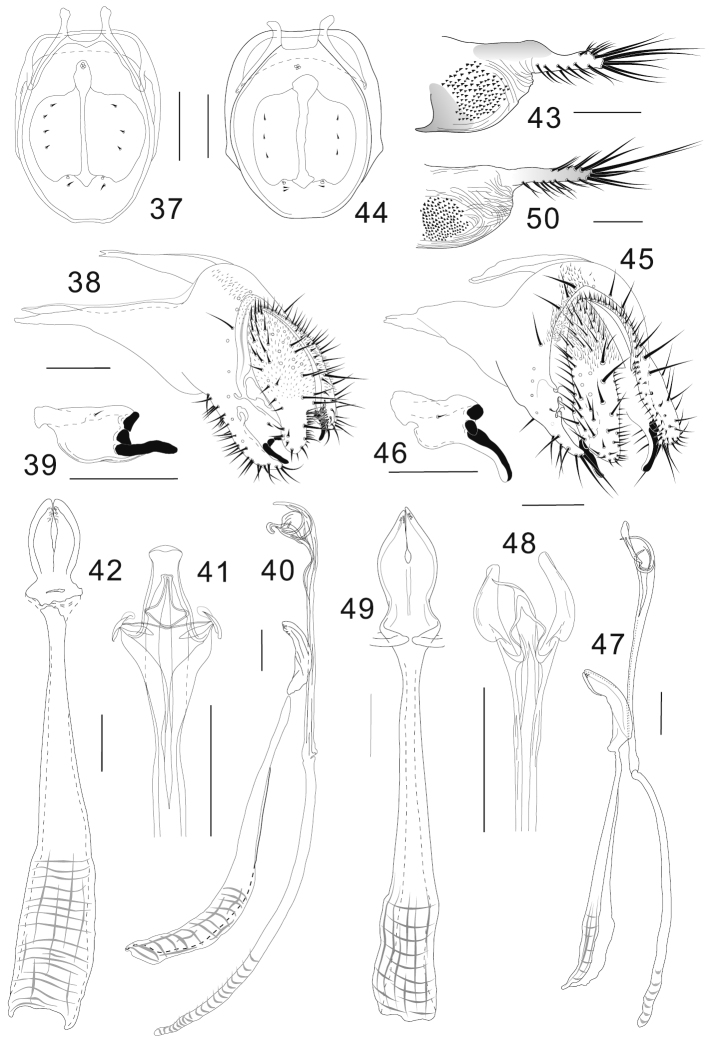
*Colocasiomyia hailini* Li & Gao, sp. n. (**37–43**) and *Colocasiomyia yini* Li & Gao, sp. n. (**44–50**). Adult males and females (paratypes) from Baihualing, Yunnan, China: **37, 44** Cibarium and clypeus (dorsal view) **38, 45** periphallic organs (posterolateral view) **39, 46** surstylus **40, 47** phallic organs (lateral view) **41, 48** apical part of aedeagus (ventral view) **42, 49** hypandrium and parameres (ventral view) **43, 50** oviscapt (lateral view). Scale lines = 0.1 mm.

**Remarks.** The characters described to be common among the three known species of the *gigantea* group by Fartyal et al. (2013) are shared also by the four new species to be described here, except for the distance between antennal sockets larger than the socket width: the former is as large as the latter in *Colocasiomyia hailini* sp. n.

#### Colocasiomyia longifilamentata

Li & Gao sp. n.

http://zoobank.org/67051239-8955-4D5D-A7CE-9B575A4270A9

http://species-id.net/wiki/Colocasiomyia_longifilamentata

Figs 5 , 12 , 19 , 23–28

##### Type material.

Holotype ♂ (No. 000068): CHINA: Baihualing, Longyang, Baoshan, Yunnan, 1500 m (25°17.19'N, 98°47.65'E), *ex* *Rhaphidophora decursiva* inflorescence at Stage III (the male phase: stamens appearing on the surface of spadix and dehiscing to release pollen), 16.vi.2011, JJ Gao (KIZ).

Paratypes: same data as holotype (7♂, 1♀: Nos 000069–78, 290, 291, 293); same but 16–17.vi.2011 (3♂, 1♀: Nos 000349, 350, 1133, 1134); same but 12.vii.2011 (1♂: No. 000168); same but 14.vii.2011 (1♂: No. 000353); same but from laboratory rearing of eggs in infructescences of *Rhaphidophora decursiva* collected on 16.vi.2011, JJ Gao (4♂, 1♀: Nos 001597, 98, 1631–33); from laboratory rearing of eggs in infructescences of *Rhaphidophora decursiva* collected from Yixiang, Simao, Pu’er, Yunnan, 1250 m (22°41.19'N, 101°7.77'E), on 12–13.xii.2012, JJ Gao and Z Fu (5♂, 5♀: Nos 001523, 26, 27–29, 31–33, 76, 77) (KIZ, SEHU).

##### Description.

**Adult male.** Head: Supracervical setae 12–15 per side. Dorsomedial arm of tentorial apodeme approximately 1/2 as long as dorsolateral arm. Eye red, somewhat roundish, with very sparse interfacetal setulae. Frontal vitta mat, black. First flagellomere not concave on inner margin. Facial carina trapeziform, medially wider than twice width of first flagellomere, as long as pedicel and first flagellomere combined. Palpus convex on ventrodistal portion. Cibarial posterior sensilla minute, 2 or 3 per side (Fig. 23). Labellum with 22 pseudotracheae per side.

Thorax: Scutum and scutellum glossy, blackish brown to black; thoracic pleura glossy, blackish brown. Acrostichal setulae in 6 rows.

Wing: Veins yellow. Halter grayish brown except for grayish yellow stalk.

Legs: Foreleg second tarsomere with 8–11 pegs (Figs 5, 12). Foreleg coxa large, with approximately 8 long setae on underside near attachment to trochanter. Small preapical dorsal setae present only on tibiae of hindlegs.

Abdomen: Tergites glossy, entirely blackish brown; II to VI+VII each bearing setulae and setae in approximately 3–4 transverse rows; setae of posteriormost row largest. Sternites pale brown to brown; VI posteriorly not bilobed.

Terminalia (Figs 24–27): Epandrium pubescent except for anterior margin, antero- and postero-ventral portion and large apodeme lobe, with 6–7 setae per side near posterior margin; anteroventral portion of epandrium curved inward, apically articulated to lateral arm of hypandrium; posteroventral lobe well developed, narrowly prolonged, scabbard-like, apically with a large peg, latero-ventrally with 12–13 short to moderate setae, dorso-subbasally with 2 very long, apically diverged setae extending almost beyond its tip, and 2 shorter ones (Fig. 24). Cercus crescent, pubescent on dorsal 2/3, with approximately 31 long setae, including 3 distinctly longer ones: 2 on ventral apex and 1 on subventral portion (Fig. 24). Membrane between epandrium and cercus pubescent dorsally to laterally. Surstylus entirely narrow, downward elongated, with only trichoid setae: 2 long at apex, 1 long on submedial inner surface, and 2 small setulae on subapical outer surface (Figs 24, 25). Median piece of 10th sternite somewhat anchor-shaped in posterior view, medially forming longitudinal ridge, laterally with broad flank. Paramere somewhat blade-like in lateral view, apically with 5 minute sensilla along edge (Figs 26, 27). Aedeagus nearly entirely separated into a pair of lateral lobes ventrally connected by subbasally to subapically densely pubescent membrane, slightly curved ventrad subapically, somewhat pointed apically; apodeme proceeding nearly along aedeagal axis, shorter than aedeagus, but longer than its 1/2; aedeagal basal processes connecting dorsobasal corners of aedeagus and lateral arms of hypandrium (Fig. 26).

Measurements (holotype / range in 6♂ paratypes, in mm): BL (straight distance from anterior edge of pedicel to tip of abdomen) = 2.76 / 2.45–2.75, ThL (medial distance from anterior notal margin to apex of scutellum) = 1.29 / 1.17–1.37, WL (distance from humeral cross vein to wing apex) = 2.49 / 2.31–2.74, WW (maximum wing width) = 1.08 / 0.96–1.07.

Indices (holotype / range in 6♂ paratypes): FW/HW (frontal width / head width) = 0.55 / 0.55−0.57, ch/o (maximum width of gena / maximum diameter of eye) = 0.52 / 0.44−0.63, prorb (proclinate orbital seta / posterior reclinate orbital seta in length) = 1.55 / 1.34–1.67, rcorb (anterior reclinate orbital seta / posterior reclinate orbital seta in length) = 0.49 / 0.38–0.56, orbito (distance between proclinate and posterior reclinate orbital setae / distance between inner vertical and posterior reclinate orbital setae) = 0.71 / 0.70−0.83, vb (subvibrissal seta / vibrissa in length) = 0.33 / 0.24–0.39, dcl (anterior dorsocentral seta / posterior dorsocentral seta in length) = 0.51 / 0.53–0.68, presctl (prescutellar seta / posterior dorsocentral seta in length) = 0.46 / 0.45–0.54, sctl (basal scutellar seta / apical scutellar seta in length) = 0.71 / 0.63–0.74, sterno (anterior katepisternal seta / posterior katepisternal seta in length) = 0.72 / 0.68–0.80, mid katepisternal seta indistinguishable from the other fine setae, dcp (distance between ipsilateral dorsocentral setae / distance between anterior dorsocentral setae) = 1.07 / 0.85−1.31, sctlp (distance between ipsilateral scutellar setae / distance between apical scutellar setae) = 1.10 / 0.94−1.39, C (2nd costal section between subcostal break and R_2+3_ / 3rd costal section between R_2+3_ and R_4+5_) = 2.56 / 2.28−2.75, 4c (3rd costal section between R_2+3_ and R_4+5_ / M_1_ between r-m and dm-cu) = 0.81 / 0.76−0.98, 4v (M_1_ between dm-cu and wing margin / M_1_ between r-m and dm-cu) = 1.44 / 1.37−1.70, 5× (CuA_1_ between dm-cu and wing margin / dm-cu between M_1_ and CuA_1_) = 0.88 / 0.75−0.82, ac (3rd costal section between R_2+3_ and R_4+5_ / distance between distal ends of R_4+5_ and M_1_) = 3.44 /2.81–4.13, M (CuA_1_ between dm-cu and wing margin / M_1_ between r-m and dm-cu) = 0.16 / 0.14–0.19, C3F (length of heavy setation in 3rd costal section / length of 3rd costal section) = 0.79 / 0.74–0.83.

**Female.** Head, thorax, wing and legs as in male.

Terminalia: Tergite VII mid-dorsally not constricted; VIII entirely pubescent, with 5 setae in transverse (against body axis) row on discolored posteroventral portion. Oviscapt distally narrowing; distal narrow portion as long as proximal, broad portion, with approximately 15, 6 and 3 trichoid ovisensilla per side on ventral, dorsal and apical margins, respectively, and a tiny, peg-like ovisensillum near dorsosubapical margin (Fig. 28).

Measurements (range in 5♀ paratypes, in mm): BL = 2.66–3.30, ThL = 1.33–1.47, WL = 2.40–2.90, WW = 1.03–1.21.

Indices (range in 5♀ paratypes): FW/HW = 0.55−0.60, ch/o = 0.51−0.59, prorb = 1.35–1.56, rcorb = 0.42−0.78, orbito = 0.65−0.80, vb = 0.28−0.39 (4♀), dcl = 0.46−0.63, presctl = 0.45–0.56, sctl = 0.65−0.74 (4♀), sterno = 0.44−0.77, dcp = 0.92−1.00, sctlp = 1.04−1.20, C = 2.21−2.68, 4c = 0.79−0.90, 4v = 1.44−1.53, 5x = 0.68−0.97, ac = 2.73−3.59, M = 0.17–0.19, C3F = 0.78–0.86.

##### Egg.

Filaments 2, approximately 1.8–2.4 times as long as length of egg body.

##### Etymology.

The specific name “*longifilamentata*” refers to the long filaments of egg.

##### Distribution.

China (Yunnan).

##### Remarks.

Although this species closely resembles *Colocasiomyia rhaphidophorae* in the external morphology and structures of male and female terminalia, it can be easily distinguished from the latter by the epandrium having several setae on the dorsal to lateral portion (Fig. 24) (*Colocasiomyia rhaphidophorae* lacking setae there).

#### Colocasiomyia longivalva

Li & Gao sp. n.

http://zoobank.org/01776174-A214-4450-8AD6-8B17016CAA15

http://species-id.net/wiki/Colocasiomyia_longivalva

Figs 6 , 13 , 20 , 29–36

##### Type material.

Holotype ♂ (No. 000079): CHINA: Baihualing, Longyang, Baoshan, Yunnan, 1500 m (25°17.19'N, 98°47.65'E), *ex* *Rhaphidophora decursiva* inflorescence at Stage III, 17.vi.2011, JJ Gao (KIZ).

Paratypes: same data as holotype but 16.vi.2011 (2♂, 8♀: Nos 000080–87, 171, 172); same but 15.vi.2011 (1♂: No. 000170) (KIZ, SEHU).

##### Description.

**Adult male.** Head: Supracervical setae 13 per side. Dorsomedial arm of tentorial apodeme 1/3 as long as dorsolateral arm. Eye red, somewhat roundish, with very sparse interfacetal setulae. Frontal vitta mat, black. First flagellomere not concave on inner margin. Facial carina trapeziform, medially wider than twice width of first flagellomere, as long as pedicel and first flagellomere combined. Palpus convex on ventrodistal portion. Cibarial posterior sensilla minute, 1 per side (Fig. 29). Labellum with 20 pseudotracheae per side.

Thorax: Scutum and scutellum glossy, black; thoracic pleura glossy, blackish brown. Acrostichal setulae in 6 rows.

Wing: Veins yellow. Halter grayish brown except for grayish yellow stalk.

Legs: Foreleg second tarsomere with 8–10 pegs (Figs 6, 13). Foreleg coxa large, with approximately 8 long setae on underside near attachment to trochanter. Small preapical dorsal setae present only on tibiae of hindlegs.

Abdomen: Tergites glossy, entirely dark brown; II to VI+VII each bearing setulae and setae irregularly arranged; setae of posteriormost row largest. Sternites grayish yellow; VI posteriorly not bilobed.

Terminalia (Figs 30–35): Epandrium notched above insertion of ventral lobe, pubescent except for anterolateral margin to ventral portion and ventral lobe, with 7–8 setae per side along ventral margin of ventral lobe and approximately 10 setae (including 2 thickest ones located just above and 2 shortest ones just on subventral notch) along posterior margin; anteroventral portion curved inward, apically articulated to lateral arm of hypandrium (Figs 30, 31); ventral lobe prolonged like rod, apically with grooved, finger-like peg (Figs 30–32). Surstylus basally articulated to inner, basal corner of epandrial ventral lobe, 1/3 as long as epandrial ventral lobe, distally nearly parallel with epandrial ventral lobe, with 2 trichoid setae apically and 2 trichoid, thinner setae on ventral, subapical surface (Figs 31, 33). Cercus large, somewhat rhombic, wider than 1/2 its (dorsoventral) height, pubescent on dorsal 2/3, with approximately 33 setae mostly distributed near posterior margin, including slightly prominent one at caudoventral apex (Fig. 30). Membrane between epandrium and cercus pubescent dorsally to laterally. Tenth sternite folded into two lateral lobes connected with each other caudodorsally; lateral lobe triangularly extended anterodorsally, fused with membrane between epandrium and cercus. Hypandrium narrow plate-like, posteriorly T-shaped, with lateral arms fused to membranous, aedeagal basal processes (Figs 34, 35). Parameres long, coalescent to hypandrium, triangular in ventral view, apically with 4 minute sensilla arranged in a row, basally fused to each other (Figs 34, 35). Aedeagus nearly entirely separated into a pair of lateral lobes, pubescent basally, bent ventrad subapically, narrowly pointed at apex; aedeagal apodeme rod-like, 1/2 as long as aedeagus (Fig. 34); aedeagal basal processes membranous, connecting dorsobasal corners of aedeagus and lateral arms of hypandrium.

Measurements (holotype / range in 3♂ paratypes, in mm): BL = 3.30 / 2.40−3.20, ThL = 1.40 / 1.18−1.42, WL = 2.80 / 2.50−2.88, WW =1.09 / 1.03−1.20.

Indices (holotype / range in 3♂ paratypes): FW/HW = 0.49 / 0.48−0.50, ch/o = 0.51 / 0.53−0.54, prorb = 1.04 / 1.25−1.49, rcorb = 0.44 / 0.44−0.50, orbito = 0.74 / 0.56−0.66, vb = 0.47 / 0.38−0.50, dcl = 0.50 / 0.51−0.59, presctl = 0.50 / 0.51−0.59, sctl = 0.91 / 0.65−0.77, sterno = 0.65 / 0.56−0.66, mid katepisternal seta indistinguishable from other fine setae, dcp = 1.08 / 0.86−1.07, sctlp = 1.23 / 1.10−1.26, C = 1.76 / 1.79−1.83, 4c = 1.16 / 1.18−1.19, 4v = 1.83 / 1.85−1.95, 5x = 0.98 / 0.99−1.18, ac = 4.54 / 3.84−4.08, M = 0.14 / 0.15−0.17, C3F = 0.83 / 0.84−0.88.

**Female.** Head, thorax, wing and legs as in male.

Terminalia: Tergite VII mid-dorsally not constricted; VIII pubescent nearly entirely, with 3−4 setae in a transverse row on unpubescent medio-posterior portion. Oviscapt distal narrow portion twice as long as proximal, broad portion, with approximately 18, 6 and 3 trichoid ovisensilla per side on ventral, dorsal and apical margins, respectively, and tiny, peg-like ovisensillum near mid-dorsal margin (Fig. 36).

Measurements (range in 8♀ paratypes, in mm): BL = 2.80−3.21 (7♀), ThL = 1.26−1.47, WL = 2.67−2.90, WW =1.08−1.26.

Indices (range in 8♀ paratypes): FW/HW = 0.49−0.52, ch/o = 0.49−0.54, prorb = 0.97−1.25, rcorb = 0.34−0.57, orbito = 0.56−0.73, vb = 0.43−0.64, dcl = 0.49−0.56, presctl = 0.49−0.56, sctl = 0.64−0.87 (7♀), sterno = 0.63−0.87, dcp = 0.82−1.06, sctlp = 1.10−1.31, C = 1.78−2.06, 4c = 1.05−1.23, 4v = 1.81−1.99, 5x = 0.88−1.18, ac = 3.55−4.36, M = 0.14−0.18, C3F = 0.84−0.89.

##### Distribution.

China (Yunnan).

##### Etymology.

Pertaining to the long oviscapt valva.

##### Remarks.

Adults of this species were very rarely captured from inflorescences of *Rhaphidophora decursiva*. So far we have never get any adult of this species by laboratory rearing from the host inflorescences/infructescences. This species is distinguished from the other members of the *gigantea* group: epandrium somewhat notched above insertion of ventral lobe; epandrial ventral lobe prolonged like rod, apically with grooved, finger-like peg (Figs 30–32); surstylus 1/3 as long as epandrial ventral lobe, distally nearly parallel with the latter (Figs 31, 33); paramere large, as long as hypandrium (Figs 34, 35); distal narrow portion of oviscapt twice as long as proximal, broad portion (Fig. 36).

#### Colocasiomyia hailini

Li & Gao sp. n.

http://zoobank.org/8F467F73-9310-499B-875B-AB023BCD6992

http://species-id.net/wiki/Colocasiomyia_hailini

Figs 7 , 14 , 21 , 37–43

##### Type material.

Holotype ♂ (No. 001641): CHINA: Baihualing, Longyang, Baoshan, Yunnan, 1500 m (25°17.27'N, 98°48.7'E), *ex* *Rhaphidophora decursiva* inflorescence at Stage III, 29.vi.2006, JT Yin (KIZ).

Paratypes: same data as holotype (11♂, 27♀: Nos 001634–40, 42–49, 51–73); same but (25°17.19'N, 98°47.65'E) (2♂, 2♀: Nos 001674, 75, 1135, 1136), 19.vi.2011, JJ Gao; same but (25°17.19'N, 98°47.65'E) (4♂: Nos 000355–58), 12–15.vii.2011, JJ Gao; same but (25°17.19'N, 98°47.65'E) (2♂, 2♀: Nos 001298–301), from laboratory rearing of eggs in infructescences of *Rhaphidophora decursiva* collected on 3–9.viii.2012, JJ Gao, Z Fu, NN Li, JM Chen and SS Li; Yixiang, Simao, Pu’er, Yunnan (22°41.19'N, 101°7.77'E) (2♂, 1♀: Nos 001448, 50, 1517), from laboratory rearing of eggs in infructescences of *Rhaphidophora decursiva* collected on 12–13.xii.2012, JJ Gao and Z Fu (KIZ, SEHU).

##### Description.

**Adult male.** Head: Supracervical setae approximately 7 per side. Dorsomedial arm of tentorial apodeme 1/3 as long as dorsolateral arm. Eye red, somewhat roundish, with very sparse interfacetal setulae. Frontal vitta mat black. First flagellomere concave on inner margin. Facial carina trapeziform, medially wider than twice width of first flagellomere, as long as pedicel and first flagellomere combined. Palpus convex on ventrodistal portion. Cibarial posterior sensilla minute, 1 per side (Fig. 37). Labellum with 11 pseudotracheae per side.

Thorax: Scutum and scutellum glossy, blackish brown to black; thoracic pleura glossy, blackish brown. Acrostichal setulae in 4 rows.

Wing: Veins yellow. Halter grayish brown except for grayish yellow stalk.

Legs: Foreleg second tarsomere with 6 pegs (Figs 7, 14). Foreleg coxa large, with approximately 15 long setae on underside near attachment to trochanter. Small preapical dorsal setae present only on tibiae of hindlegs.

Abdomen: Tergites glossy, entirely dark brown; II to VI+VII each bearing setulae and setae in approximately 3 transverse rows; setae of posteriormost row largest. Sternites pale brown to brown; VI posteriorly not bilobed.

Terminalia (Figs 38–42): Epandrium pubescent dorsomedially only, with 6 setae per side near posterior margin, 15–16 setae per side on ventral portion and 23–24 setae as thick as upper cercal ones along ventral margin of ventral lobe; apodeme well developed into distally tapering, triangular extension strongly projected anteriad, twice as long as epandrial width, broadly sclerotized along dorsal and ventral margins (Fig. 38); anteroventral portion of epandrium curved inward, apically articulated to lateral arm of hypandrium. Surstylus broad, basally narrowly fused to basal corner of epandrial ventral lobe, dorsally broadly sclerotized, with 1 short, trichoid seta on upper medial portion of outer surface and 3 large, peg-like prensisetae on distal margin; lowest prensiseta nearly as long as width of surstylus, curved inwards and slightly downwards, especially in distal 1/3, narrowly edged by caudoventral portion of surstylus only along its basal portion (Figs 38, 39). Cercus semilunar, narrower than 1/2 its (dorsoventral) height, pubescent on dorsal 2/3, with approximately 48 setae (including prominent one twice as long as others) all over and approximately 45 setulae on caudoventral, inner margin; ventral slightly incurved lobe 1/3–1/4 of cercal height (Fig. 38). Membrane between epandrium and cercus pubescent dorsally to laterally. Median piece of 10th sternite cordiform in posterior view, moderately sclerotized; lateral piece somewhat cuneiform, narrowing anteriad, connected to inner, basal corner of epandrial ventral lobe with membranous tissue. Hypandrium long, narrow, plate-like, anteriorly widened (Fig. 42). Parameres somewhat semilunar in ventral view, basally fused to each other, apically with 5 or 6 minute sensilla in small medioapical patch (Figs 40, 42). Aedeagus nearly entirely separated into a pair of lateral lobes, nearly straight, apically trilobed; lobes curved ventrad and connected with each other by tendon-like membranous structures (Figs 40, 41); apodeme rod-like, arched in lateral view, longer than aedeagus (Fig. 40).

Measurements (holotype / range in 10♂ paratypes, in mm): BL = 2.01 / 1.85−2.14, ThL = 0.86 / 0.76−0.94, WL = 1.79 / 1.59−1.89, WW = 0.79 / 0.72−0.85.

Indices (holotype / range in 10♂ paratypes): FW/HW = 0.54 / 0.53−0.56, ch/o = 0.48 / 0.46−0.55, prorb = broken / 1.08 (1♂), rcorb = 0.61 / 0.56−0.59 (6♂), orbito = 0.86 / 0.63−0.85, vb = 0.31 / 0.30−0.36, dcl = 0.52 / 0.54−0.62 (6♂), presctl = 0.38 / 0.30−0.39 (9♂), sctl = 0.75 / 0.59−0.77 (9♂), sterno = 0.75 / 0.72−0.94, mid katepisternal seta indistinguishable from other fine setae, dcp = 0.92 / 0.82−1.02, sctlp = 1.00 / 0.92−1.12, C = 1.90 / 1.72−2.12, 4c = 1.18 / 0.89−1.21, 4v = 1.79 / 1.28−1.75, 5x = 1.15 / 0.94−1.17, ac = 3.56 /3.04−3.59, M = 0.19 / 0.19−0.21, C3F = 0.48 / 0.35−0.53.

**Female.** Head, thorax, wing and legs as in male.

Terminalia: Tergite VII mid-dorsally not constricted; VIII pubescent nearly entirely, with 4–5 setae in a longitudinal row on unpubescent, discolored, posteroventral portion. Oviscapt distal elongation constricted dorsally at basal 1/3, apically somewhat truncate, with 6, 9 and 3 trichoid ovisensilla per side on distal 1/3 of dorsal margin, entire ventral margin, and at apex, respectively (Fig. 43).

Measurements (range in 10♀ paratypes, in mm): BL = 2.43−2.95, ThL = 0.89−1.22, WL = 1.90−2.48, WW = 0.85−1.07.

Indices (range in 10♀ paratypes): FW/HW = 0.55−0.59, ch/o = 0.50−0.62, prorb broken, rcorb = 0.40−0.52 (3♀), orbito = 0.68−0.91, vb = 0.29−0.35, dcl = 0.43−0.57 (6♀), presctl = 0.29−0.37 (7♀), sctl = 0.69−0.77 (9♀), sterno = 0.74−0.91, dcp = 0.90−1.09, sctlp = 1.00−1.12, C = 1.83−2.23, 4c = 1.00−1.14, 4v = 1.50−1.86, 5x = 0.87−1.19, ac = 3.28−4.00, M = 0.16−0.20, C3F = 0.38−0.59.

##### Distribution.

China (Yunnan).

##### Etymology.

The specific name “*hailini*” is a patronym in honor of the emeritus Professor Hai-lin Wang of the Southwest Forestry University, China.

##### Remarks.

This species resembles the following species, *Colocasiomyia yini* sp. n., in overall outer morphology, but can be distinguished from the latter by 1) C3F < 2/3 (C3F ≥ 2/3 in *yini* sp. n.); 2) lowest, peg-like prensiseta on distal margin of surstylus curved inwards and slightly downwards, especially in distal 1/3, narrowly edged by caudoventral portion of surstylus only along its basal portion (Fig. 39) [in *Colocasiomyia yini* sp. n., lowest peg-like prensiseta strongly curved downwards in distal half, narrowly edged by caudoventral portion of surstylus along its whole length (Figs 46)]; 3) ventral slightly incurved lobe of cercus 1/3–1/4 of cercal height (Fig. 38) [in *Colocasiomyia yini*, ventral lobe of cercus approximately 1/2 of cercal height (Fig. 45)]; 4) distal, narrow part of oviscapt constricted dorsally at basal 1/3 (Fig. 43) [in *Colocasiomyia yini* sp. n., distal, narrow part of oviscapt almost even-edged (Fig. 50)].

#### Colocasiomyia yini

Li & Gao sp. n.

http://zoobank.org/0A22EA8C-6EF6-48DF-B46C-B946CEE327E9

http://species-id.net/wiki/Colocasiomyia_yini

Figs 8 , 15 , 22 , 44–50

##### Type material.

Holotype ♂ (No. 000159): CHINA: Baihualing, Longyang, Baoshan, Yunnan, 1500 m (25°17.19'N, 98°47.65'E), *ex* *Rhaphidophora decursiva* inflorescence at Stage III, 12.vii.2011, JJ Gao (KIZ).

Paratypes. Same data as holotype (3♂, 2♀: Nos 000160, 165-167, 178); same but 16.vi.2011 (1♂, 2♀: Nos 000161-163); same but (25°17.27'N, 98°48.7'E), 29.vi.2006 (1♀: No. 001650), JT Yin; same but 13.vii.2011 (1♀: No. 000164); same but 14.vii.2011 (1♂: No. 000169); same but from laboratory rearing of eggs in infructescences of *Rhaphidophora decursiva* collected on 23–24.ix.2012, JJ Gao, Z Fu and JM Chen (1♀: No. 001185); from laboratory rearing of eggs in infructescences of *Rhaphidophora decursiva* collected from Yixiang, Simao, Pu’er, Yunnan (22°41.19'N, 101°7.77'E) on 12–13.xii.2012, JJ Gao and Z Fu (1♀: No. 001586) (KIZ, SEHU).

##### Description.

**Adult male.** Head: Supracervical setae 11 per side. Dorsomedial arm of tentorial apodeme 1/3 as long as dorsolateral arm. Eye red, somewhat roundish, with very sparse interfacetal setulae. Frontal vitta mat black. First flagellomere not concave on inner margin. Facial carina trapeziform, medially wider than twice width of first flagellomere, as long as pedicel and first flagellomere combined. Palpus convex on ventrodistal portion. Cibarial posterior sensilla minute, 1 or 2 per side (Fig. 44). Labellum with 11 pseudotracheae per side.

Thorax: Scutum and scutellum glossy, black; thoracic pleura glossy, blackish brown. Acrostichal setulae in 4 rows.

Wing: Veins yellow. Halter dark brown except for grayish yellow stalk.

Legs: Foreleg second tarsomere with 6 pegs (Figs 8, 15). Foreleg coxa large, with approximately 10 long setae on underside near attachment to trochanter. Small preapical dorsal setae present only on tibiae of hindlegs.

Abdomen: Tergites glossy, entirely dark brown; II to VI+VII each bearing setulae and setae in approximately 3 transverse rows; setae of posteriormost row largest. Sternites II–V pale brown; VI blackish brown, and bilobed posteriorly.

Terminalia (Figs 45–49): Epandrium with 6 setae per side from lateral portion to middorsal, posterior margin, 14 setae per side in ventral portion and 15–16 setae as thick as upper cercal ones along ventral margin of ventral lobe; apodeme well developed into distally tapering, triangular extension strongly projected anteriad, twice as long as epandrial width, broadly sclerotized along dorsal and ventral margins (Fig. 45); anteroventral portion of epandrium curved inward, apically articulated to lateral arm of hypandrium (Fig. 45). Surstylus basally fused to basal corner of epandrial ventral lobe, dorsally broadly sclerotized, with 1 short, trichoid seta on upper medial portion, and 3 large, peg-like prensisetae on distal margin; lowest prensiseta slightly longer than width of surstylus, strongly curved downwards in distal half, narrowly edged by caudoventral portion of surstylus along its whole portion (Figs 45, 46). Cercus oblong, narrower than 1/2 its (dorsoventral) height, pubescent on dorsal 1/2, with approximately 58 setae (including one distinctively longer than others) all over and approximately 26 setulae on caudoventral, inner margin; ventral lobe approximately 1/2 of cercal height (Fig. 45). Membrane between epandrium and cercus pubescent dorsally to laterally. Median piece of 10th sternite rhombic in posterior view, moderately sclerotized; lateral piece somewhat cuneiform, narrowing anteriad, connected to inner, basal corner of epandrial ventral lobe with membranous tissue. Hypandrium long, narrow, plate-like, anteriorly widened, posteriorly T-shaped, with lateral arms fused to membranous aedeagal basal processes (Figs 47, 49). Parameres somewhat semilunar in ventral view, basally fused to each other, apically with 6 minute sensilla in small patch (Figs 47, 49). Aedeagus nearly entirely separated into a pair of lateral lobes, slightly bent, apically trilobed; median lobe curved ventrad and connected with lateral ones by tendon-like membranous structures (Figs 47, 48); apodeme rod-like, arched in lateral view, as long as aedeagus (Fig. 47); aedeagal basal processes membranous, connecting dorsobasal corners of aedeagus and posterolateral expansions of hypandrium.

Measurements (holotype / range in 5♂ paratypes, in mm): BL = 2.66 / 2.20−2.56 (4♂), ThL = 1.12 / 0.90−1.05, WL = 2.27 / 1.87−2.27, WW =1.03 / 0.85−1.00.

Indices (holotype / range in 5♂ paratypes): FW/HW = 0.53 / 0.50−0.56, ch/o = 0.54 / 0.47−0.59, prorb = 1.04 / 0.97−1.20, rcorb = 0.42/ 0.48−0.55, orbito = 0.58 / 0.56−0.68, vb = 0.46 / 0.40−0.52, dcl = 0.50 / 0.47−0.50, presctl = 0.34 / 0.24−0.37, sctl = 0.62 / 0.57−0.69 (4♂), sterno = 0.79 / 0.79−1.21 (3♂), mid katepisternal seta indistinguishable from other fine setae, dcp = 0.88 / 0.80−0.90, sctlp = 1.15 / 0.90−1.03, C = 2.35 / 2.04−2.52, 4c = 0.96 / 0.95−1.07, 4v = 1.67 / 1.63−1.92, 5x = 1.02 / 1.06−1.27, ac = 3.75 / 3.00−3.68, M = 0.15 / 0.16−0.18, C3F = 0.77 / 0.69−0.80.

**Female.** Head, thorax, wing and legs as in male.

Terminalia: Tergite VII mid-dorsally not constricted; VIII pubescent nearly entirely, with 5−6 setae in a transverse row on unpubescent, discolored, posteroventral portion. Oviscapt distal elongation almost smooth on dorsal margin, with 5, 7 and 3 trichoid ovisensilla per side on distal 1/2 of dorsal margin, entire ventral margin and at apex, respectively, of distal elongation (Fig. 50).

Measurements (range in 5♀ paratypes, in mm): BL = 2.05−2.86 (4♀), ThL = 0.93−1.20, WL = 2.20−2.60, WW = 0.86−1.05.

Indices (range in 5♀ paratypes): FW/HW = 0.53−0.56, ch/o = 0.43−0.60, prorb = 0.88−1.11, rcorb = 0.28−0.51, orbito = 0.60−0.70, vb = 0.29−0.45, dcl = 0.46−0.52, presctl = 0.26−0.34, sctl = 0.50−0.65 (4♀), sterno = 0.76−0.83 (4♀), dcp = 0.81−0.84, sctlp = 0.85−1.13, C = 2.20−2.34, 4c = 0.96−1.03, 4v = 1.67−1.92, 5x = 0.91−1.23, ac = 3.12−4.76, M = 0.13−0.18, C3F = 0.65−0.74.

##### Distribution.

China (Yunnan).

##### Etymology.

In honor of Mr Jian-Tao Yin of the Xishuangbanna Tropical Botanical Garden, Chinese Academy of Sciences.

##### Remarks.

Much fewer adults of this species were collected from inflorescences of *Rhaphidophora decursiva* in comparison to *Colocasiomyia hailini* sp. n. and *Colocasiomyia longifilamentata* sp. n. Breeding of this species on *Rhaphidophora decursiva* was confirmed by laboratory rearing of eggs laid on inflorescences of the host plant. See the Remarks for *Colocasiomyia hailini* sp. n. with respect to morphological differences from it.

### A key to species of the *Colocasiomyia gigantea* species group

In this key, the numbers of figures of Fartyal et al. (2013) are given in double quotation marks.

**Table:** 

1	Labellum with 16 pseudotracheae per side. Aedeagal apodeme distinctly longer than aedeagus (“Fig. 4C”). Distal, narrow part of oviscapt narrowing and gently curved ventrad, apically arrowhead-shaped (“Fig. 4E”)	*Colocasiomyia scindapsae* Fartyal & Toda
–	Labellum with ≤ 14 or ≥ 20 pseudotracheae per side. Aedeagal apodeme as long as or shorter than aedeagus (Figs 26, 34, 40, 47; “Figs 2F, 3D”). Distal narrow part of oviscapt truncate apically, or curved dorsad if not truncate	2
2	Labellum with 14 pseudotracheae per side. Distal, narrow part of oviscapt broadly truncate apically, much shorter than proximal, broad part (“Fig. 2H”)	*Colocasiomyia gigantea* (Okada)
−	Labellum with 11 or ≥ 20 pseudotracheae per side. Distal, narrow part of oviscapt not or only slightly truncate apically, curved dorsad apically, longer or only slightly shorter than proximal, broad part (Figs 28, 36, 43, 50)	3
3	Labellum with 11 pseudotracheae per side. Foreleg tarsomere II with 6 pegs (Figs 7, 8, 14, 15). Acrostichal setulae in 4 rows	4
−	Labellum with ≥ 20 pseudotracheae per side. Foreleg tarsomere II with ≥ 8 pegs (Figs 2–6, 9–13). Acrostichal setulae in 6 rows	5
4	Wing C3F index < 2/3. Distance between antennal sockets same as socket width. Distal, narrow part of oviscapt constricted subbasally on dorsal margin (Fig. 43)	*Colocasiomyia hailini* Li & Gao, sp. n.
−	Wing C3F index > 2/3. Distance between antennal sockets larger than socket width. Distal, narrow part of oviscapt finger-like, not constricted subbasally on dorsal margin (Fig. 50)	*Colocasiomyia yini* Li & Gao, sp. n.
5	Epandrium notched above basal corner of epandrial ventral lobe; ventral lobe prolonged like rod, apically with grooved, finger-like peg (Figs 30–32). Surstylus 1/3 as long as epandrial ventral lobe, distally nearly parallel with the latter (Figs 31, 33). Hypandrium as long as paramere (Figs 34, 35). Distal, narrow part of oviscapt twice as long as proximal, broad part (Fig. 36)	*Colocasiomyia longivalva* Li & Gao, sp. n.
–	Epandrium not notched along posterior margin; ventral lobe narrowing distally, apically with ungrooved, apically pointed peg (Fig. 24; “Fig. 3C”). Surstylus entirely narrow, elongated downward, as long as epandrial ventral lobe (Fig. 25; “Fig. 3C”). Hypandrium 3–4 times as long as paramere (Figs 26, 27; “Fig. 3D, E”). Distal, narrow part of oviscapt as long as or shorter than proximal, broad part (Fig. 28; “Fig. 3G”)	6
6	Posterior margin of epandrium without setae (“Fig. 3C”). Palpus dark, apically swollen	*Colocasiomyia rhaphidophorae* Gao & Toda
–	Posterior margin of epandrium with several setae (Fig. 24). Palpus pale, apically not swollen	*Colocasiomyia longifilamentata* Li & Gao, sp. n.

## Discussion

DNA barcoding has been innovated to facilitate works of not only taxonomists but also non-experts in specimen identification (http://ibol.org/about-us/background/). Even for expert taxonomists, it is not trivial to identify pre-imaginal stages or a mass of adult individuals of closely related species in Drosophilidae. In *Colocasiomyia*, it is known in a number of cases, including that given by the present study, that closely related species cohabit on the same host plant. To study mechanisms for cohabitation in such systems, DNA barcoding approaches should be very useful in species identification, especially for pre-imaginal stages. However, the “barcoding gap” between intra- and interspecific genetic distances based on *COI* sequences is too flimsy to validate the objective, distance-based species delimitation in the *gigantea* group. Similarly, in some previous studies, distance-based DNA barcoding based on *COI* sequences did not work well for species delimitation due to the overlapping of inter- and intraspecific distances (see DeSalle et al. 2005, and references therein). DeSalle et al. (2005) thus proposed a character-based approach to overcome the shortcomings of distance-based species identification (e.g., lacking of objective distance criterion). In the present study, both morphological characters and DNA barcodes were incorporated into species delineation in the *gigantea* group: some nucleotide sites with fixed status in the *COI* sequences were recognized as “pure” diagnostics for each species of the *gigantea* group. However, the effectiveness of such diagnoses still need to be verified by sampling more sequences from each species and strengthened by sequencing more genes.

Unlike the other *Colocasiomyia* species, all the species of the *gigantea* group breed on (*Colocasiomyia longivalva* at least visits) inflorescences/infructescences of the subfamily Monsteroideae (Araceae). All of the host plants are hemiepiphytic, bisexual-flowered climbers belonging to the *Rhaphidophora* clade (Cusimano et al. 2011, Nauheimer et al. 2012) in the Monsteroideae. *Colocasiomyia scindapsae* and *Colocasiomyia gigantea* monopolize their host plants, *Scindapsus coriaceus* and *Epipremnum pinnatum* in Sabah and Java, respectively (Fartyal et al. 2013). In this study, the five Chinese species of the *gigantea* group were found sharing the same host plant, *Rhaphidophora decursiva*. Moreover, one of them, *Colocasiomyia rhaphidophorae*, utilizes also *Rhaphidophora hookeri* as a host plant in Xishuangbanna, southern Yunnan (Fartyal et al. 2013).

The monsteroid plants as hosts of the *gigantea* group are quite different in the structure of spadix and the fruiting process from the Aroideae known as hosts of other *Colocasiomyia* species groups. Fartyal et al. (2013) have revealed that, in single-host/single-user systems, the *gigantea*-group species have evolved peculiar lifecycles, ecological traits and morphological features adaptive to characteristics of the monsteroid host plants. According to our preliminary observations, similar adaptations are also seen in the species cohabiting on *Rhaphidophora decursiva* (Li et al. unpubl. data). The discovery of such a system in which multiple species of the *gigantea* group cohabit provides an opportunity to study parallel evolution of breeding habits to achieve the cohabitation: it is known, in the *cristata* species group, that breeding habits vary according to the number and combination of cohabiting species (Carson and Okada 1980, Toda and Okada 1983, Honda-Yafuso 1983, Yafuso 1994, Takenaka 2006, Takenaka et al. 2006, Takano et al. 2012).

So far, our knowledge about the biogeography of the *gigantea* group is apparently very limited with respect to the known geographical distribution of respective host plants. *Colocasiomyia scindapsae* has been recorded only from Mt. Kinabalu (Sabah), *Colocasiomyia gigantea* from Bogor (West Java) and Solomon Is., and the others from Yunnan, China. The ranges of their host plant species are wider: *Scindapsus coriaceus* is distributed in Indonesia and Malaysia (http://gwannon.com/species/Scindapsus-coriaceus), *Rhaphidophora hookeri* in China, Myanmar and Vietnam (http://www.gwannon.com/species/Rhaphidophora-hookeri), *Rhaphidophora decursiva* in Himalaya, India and Indonesia (http://en.hortipedia.com/wiki/Raphidophora_decursiva), and *Epipremnum pinnatum* being native to New Guinea, the Malay Archipelago and the Pacific Islands (http://en.hortipedia.com/wiki/Epipremnum_pinnatum) but currently distributed almost all over the Oriental and Australasian Region (Li 1996). In addition, the *Rhaphidophora* clade consists of approximately 170 species (Tam et al. 2004), which may include many potential hosts for *Colocasiomyia* flies. It is very likely, in the future, that more species of the *gigantea* group will be discovered from different host plants, and that more distribution and host-plant records will be brought from a wider geographic range. Such information is badly needed to explore the evolution of pollination mutualisms between flies of the *gigantea* group and their monsteroid host plants.

## Supplementary Material

XML Treatment for Colocasiomyia longifilamentata

XML Treatment for Colocasiomyia longivalva

XML Treatment for Colocasiomyia hailini

XML Treatment for Colocasiomyia yini
